# An Electrochemical Immunosensor for Detection of *Staphylococcus aureus *Bacteria Based on Immobilization of Antibodies on Self-Assembled Monolayers-Functionalized Gold Electrode

**DOI:** 10.3390/bios2040417

**Published:** 2012-10-16

**Authors:** Mohamed Braiek, Karima Bekir Rokbani, Amani Chrouda, Béchir Mrabet, Amina Bakhrouf, Abderrazak Maaref, Nicole Jaffrezic-Renault

**Affiliations:** 1 Laboratoire de Physique et Chimie des Interfaces, Faculté des Sciences de Monastir, Tunisie, Avenue de l’Environnement, 5019 Monastir, Tunisia; E-Mails: amani.chrouda@yahoo.fr (A.C.); abderrazak.maaref@fsm.rnu.tn (A.M.); 2 Institut des Sciences Analytiques, UMR CNRS 5280, Université Claude Bernard-Lyon1, Bâtiment CPE, 69622 Villeurbanne Cedex, France; E-Mail: nicole.jaffrezic@univ-lyon1.fr; 3 Laboratoire d’Analyse, Traitement et Valorisation des Polluants de l’Environnement et des Produits, Faculté de Pharmacie de Monastir, Rue Avicenne 5000, Monastir, Tunisia; E-Mails: karima_rokbani@yahoo.fr (K.B.R.); mrabetbechir@yahoo.fr (B.M.); aminafdhila@yahoo.fr (A.B.)

**Keywords:** *Staphylococcus aureus*, self-assembled monolayer, immunosensor, cyclic voltammetry, electrochemical impedance spectroscopy

## Abstract

The detection of pathogenic bacteria remains a challenge for the struggle against biological weapons, nosocomial diseases, and for food safety. In this research, our aim was to develop an easy-to-use electrochemical immunosensor for the detection of pathogenic *Staphylococcus aureus *ATCC25923. The biosensor was elaborated by the immobilization of anti-*S. aureus* antibodies using a self-assembled monolayer (SAMs) of 3-Mercaptopropionic acid (MPA). These molecular assemblies were spontaneously formed by the immersion of the substrate in an organic solvent containing the SAMs that can covalently bond to the gold surface. The functionalization of the immunosensor was characterized using two electrochemical techniques: cyclic voltammetry (CV) and electrochemical impedance spectroscopy (EIS). Here, the analysis was performed in phosphate buffer with ferro/ferricyanide as the redox probe. The EIS technique was used for affinity assays: antibody-cell binding. A linear relationship between the increment in the electron transfer resistance (R_CT_) and the logarithmic value of *S. aureus* concentration was observed between 10 and 10^6^ CFU/mL. The limit of detection (LOD) was observed at 10 CFU/mL, and the reproducibility was calculated to 8%. Finally, a good selectivity *versus E. coli* and *S. epidermidis* was obtained for our developed immunosensor demonstrating its specificity towards only *S. aureus*.

## 1. Introduction

*Staphylococci* are constantly present all around us. They are most frequently found in common infections of skin, e.g., after shaving, around the nose, and in children’s scraped knees. It is also the bacteria most frequently involved in nosocomial infections. Amongst *staphylococci*, some strains have the possibility under certain conditions to produce enterotoxins responsible for food poisoning. This production also implies the presence of large quantities of germs in food [1]. Here, we will study *Staphylococcus aureus *detection because they are the species most commonly found in *staphylococcal* food poisoning [2,3].

Traditional standard microbiological culture tests are performed to detect and enumerate *S. aureus*, but these techniques are time consuming, require skilled personnel, and provide responses within 24 to 48 h [4]. The objective of this work was to develop an immunosensor for the detection of these pathogens. Many recent studies are focused on *Escherichia coli (E. coli) *and *Salmonella* bacteria detection with different transducing techniques. These include QCM [5], SPR [6], electrochemical techniques: capacitive [7] and amperometric [8] measurements, however, few studies were devoted to *S. aureus* detection [9,10,11].

In this paper, we describe an approach for the detection of this bacterium using Electrochemical Impedance Spectroscopy (EIS), given that, impedance spectroscopy is a powerful analytical technique. The application of EIS is widespread and the technique is utilized in different areas of research, such as studying the properties of chemical reactions, corrosion, and dielectric characterization, while possibly also contributing to the interpretation of electrochemical processes [12]. In the field of biosensors, it has been widely used for immunodetection [13].

The main principle for the development of such a biosensor is the functionalization of the transducer surface in order to formulate an improved biomatrix/electrode interface. Many recent works have focused on self-assembled monolayers (SAMs) of organic alkanethiols, disulfides, and sulfides, which are strongly chemisorbed on various metal surfaces such as gold. This is due to the significant affinity of sulfur atoms to the gold surface [14].

In this work, the anti-*S. aureus* antibody was covalently anchored on to the gold electrode surface through the grafting of a SAMS of 3-Mercaptopropionic acid (MPA) previously formed on the gold surface. The different steps for the immunosensor functionalization were characterized using contact angle measurements (CAM) and electrochemical measurements. EIS technique was used for affinity assays, namely, antibody-cell binding. Through modeling of Nyquist plots, a calibration curve for *S. aureus* was determined and analytical characteristics of this immunosensor were deduced and compared to those that have been previously published in the literature.

## 2. Experimental

### 2.1. Reagents

Polyclonal antibodies (developed in rabbit) against *Staphylococcus aureus* were obtained from BIOtech RDP (Sfax, Tunisia). 3-Mercaptopropionic acid (MPA), phosphate buffered saline (PBS), N-(3-dimethylaminopropyl)-N′-ethylcarbodiimide hydrochloride (EDC), N-hydroxysuccinimide (NHS), potassium ferrocyanide (K_4_Fe(CN)_6_), potassium ferricyanide (K_3_Fe(CN)_6_), ethanolamine, sulfuric acid, hydrogen peroxide (30%) were purchased from Sigma Aldrich, France. Ethanol was obtained from Fluka (purity >99%). All solutions were prepared with ultra-pure Milli-Q water.

### 2.2. Bacteria Cultivation

In the present study, *S. aureus* reference strain (ATCC25923) was used. The bacteria strain was cultured in a tryptic soy broth (TSB, (Difco)) or on TSB agar plates for 24 h at 37 °C. A high titer of bacteria suspension was prepared as follows: liquid culture media was inoculated by 100 μL of the pre-culture solution and then cultivated at 37 °C for 18–24 h. The bacterial culture was centrifuged for 5 min at 6,400 rpm and was washed twice. Finally, the culture was resuspended in sterile PBS. The determination for the rate of viable cells and bacterial concentration was performed with the spread-plate technique. The optical density (OD) of the bacterial culture was measured for the determination of bacterial growth stationary phase. The cultures of *S. aureus* strain were grown to late log phase (OD_600_ = 0.6). At the growth stationary phase, bacteria concentration does not change with time, and, subsequently, all the operations for the determination of the bacteria concentration can be performed with accuracy.

### 2.3. Instrumentation and Techniques

Electrochemical measurements were performed using a potentiostat-galvanostat Voltalab 40 with a standard three-electrode configuration. Platinum plate was used as the auxiliary electrode, saturated calomel electrode (SCE) as the reference electrode, and gold plate as the working electrode. Two electrochemical techniques were applied in this work: 

- Cyclic voltammetry was performed in 8 mM ferro/ferricyanide PBS solution at a scan rate of 100 mV/s.

- EIS is based upon inducing a disturbance in an electrochemical reaction from its steady state by applying a small excitation sinusoidal signal to the system (amplitude 10 mV; frequency range 100 mHz to 100 kHz).

- The SAMs were characterized through CAM, using a GBX Scientific Instruments (Romans-France). All measurements were made by depositing 1 µL of Milli-Q water at 24 ± 2 °C. Multiple values were recorded for each analysis. The precision of the micro-regulator was 0.33 µL for a 1 µL drop. The results were processed using Windrop++ software.

### 2.4. Elaboration of the Immunosensor

**Figure 1:**
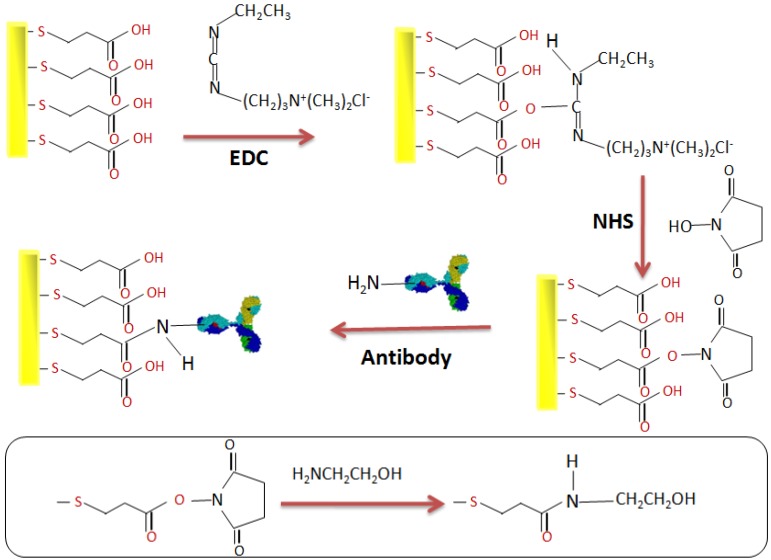
Different stages in the development of the biofilm.

The Au electrodes were ultrasonically cleaned in acetone for 10 min, followed by 5 min in a mixture of piranha solution (H_2_O_2_/H_2_SO_4_, 3/7, v/v). The electrodes were thoroughly rinsed with ultrapure water, followed by absolute ethanol, and then dried in a flow of nitrogen. Subsequently, the electrodes were incubated in a MPA (10 mM) solution made-up with ethanol for 12 h to formulate the SAMs. After the formation, the electrodes were rinsed in ethanol. Then, the terminal carboxylic acid (–COOH) groups were activated in a solution of NHS/EDC (0.1 M/0.1 M) for 1 h at room temperature. After rinsing with PBS, the electrodes were incubated for 1 h in a 0.3 mg/mL solution of anti-*S. aureus *antibodies. The terminal amine groups on the antibody enable covalent bonding to occur through the activated carboxylic functions from MPA functionalized NHS/EDC. Finally, after rinsing with PBS, the electrodes were incubated for 20 min in ethanolamine. This blocks the remaining acidic functionalities. The mechanism for this activation can be seen in Figure 1.

We studied the sensor response towards different concentrations of the *S. aureus* bacteria. For this reason, the electrodes were incubated in different aliquots corresponding to different bacterial concentrations in CFU/mL.

## 3. Results and Discussion

### 3.1. Characterization of the Functionalized Electrode

**Figure 2:**
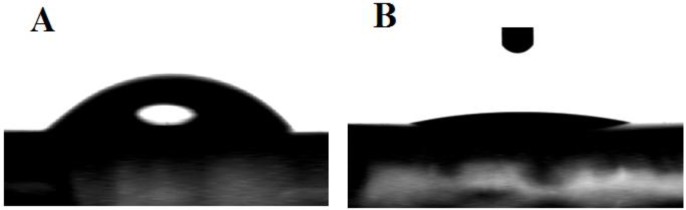
Contact angles and properties of MPA, where, (**A**) bare gold with the angle measured at 48.3 ± 1°, and (**B**) MPA monolayer with the angle measured at 7.4 ± 1°.

After thorough cleansing and immersion into MPA ethanolic solution, the gold electrode was characterized by water contact angles. The results are shown in Figure 2. Here, we observe that the contact angle decreases from 48° to 7° after SAMs deposition. This value is in agreement with results already published [15]. In this instance, when the carboxylic acid-terminated MPA monolayer is previously rinsed in ethanol, then, the observed contact angle is found to be <10°.

**Figure 3:**
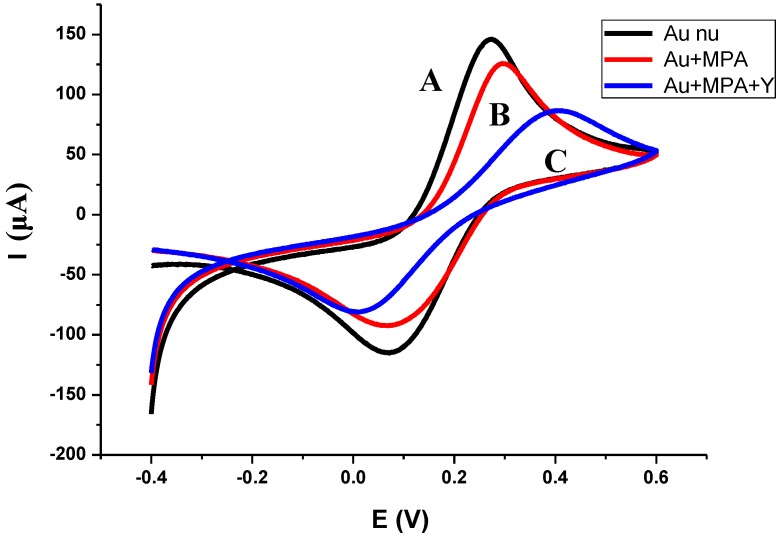
CV of (**A**) bare gold electrode, (**B**) MPA functionalization, and (**C**) antibody binding. The measurements were made in the presence of Fe(CN_6_)^3–/4–^ as the redox probe with scan rate of 100 mV/s.

**Figure 4:**
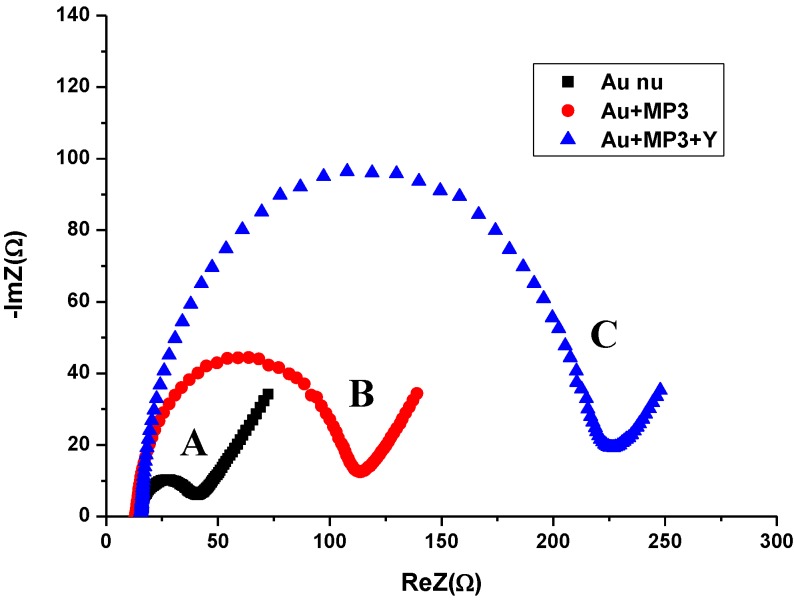
Nyquist plots of impedance spectra for (**A**) bare gold electrode, (**B**) MPA functionalization, and (**C**) antibody binding. The EIS were recorded in the presence of Fe(CN_6_)^3–/4–^ at a frequency range between 0.1 Hz and 100 kHz.

For electrochemical measurements, the CV was recorded before and after the deposition of the MPA monolayer. In Figure 3, the Fe(II)/Fe(III) redox peaks have decreased after the functionalization of the MPA monolayer. This can be attributed to the decrease of the electron transfer rate that was created by the compactness of the formulated SAMs. After antibody binding, the redox peaks decrease even more due to the decrease of the electron transfer rate. This was due to an increase of the biolayer thickness that was developed on the gold surface.

The Faradaic impedance measurements are in good agreement with CV measurements as the diameter of semicircles for the Nyquist diagrams increase significantly after the different stages for the elaboration of the immunosensor (Figure 4).

### 3.2. Impedance Measurements for the Detection of S. aureus Bacteria

**Figure 5:**
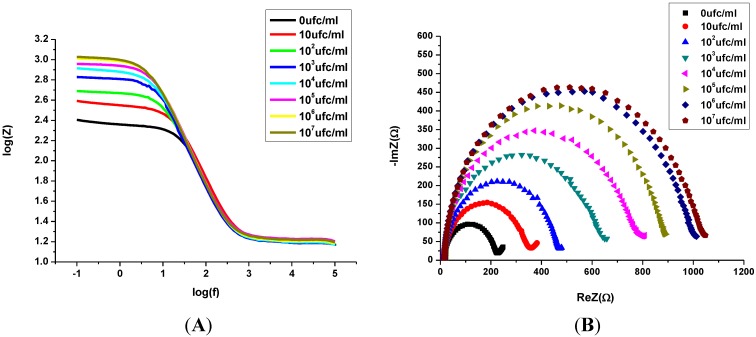
Electrochemical detection of (**A**) Bode plot, and (**B**) Nyquist plot of impedance spectra obtained from the increasing concentration of *S. aureus* (ATCC25923) from 10 to 10^7^ CFU/mL in PBS and Fe(CN_6_)^3–/4–^.

The impedance measurement for the direct detection of bacterial cells is generally known to have improved significance by analysis with a Bode plot. This technique is suitable for studying the direct relationship of impedance with the frequency [16]. Here, the binding ability of bacteria and the bioreceptor was then verified through the EIS technique. In Figure 5, we show (A) the Bode plot, and (B) the Nyquist diagram that were obtained after incubation in an increasing concentration of the target bacteria. Here, the charge transfer resistance (R_CT_) increases gradually as the bacteria concentration increases after consecutive incubations. This was made from 10 CFU/mL to 10^7^ CFU/mL.

**Figure 6:**
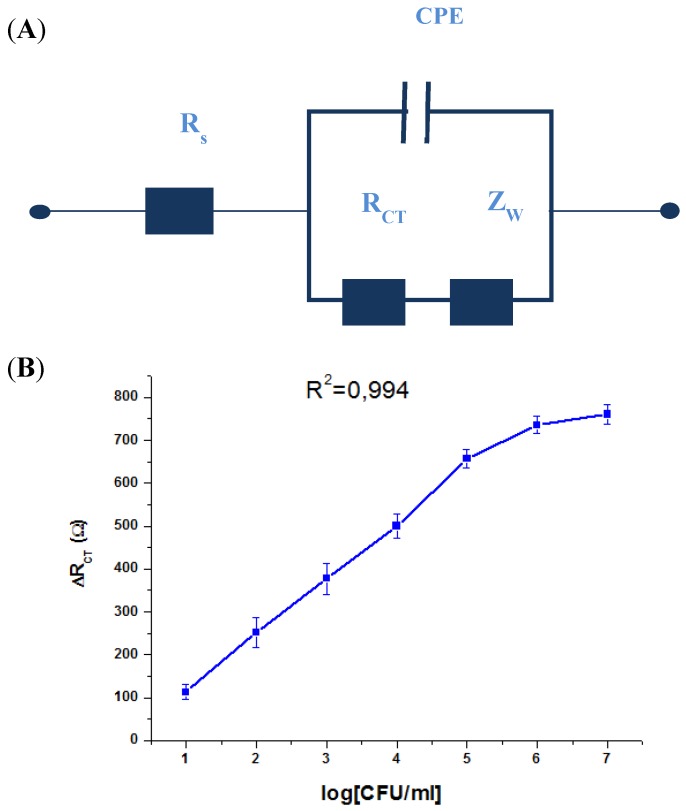
(**A**) The applied equivalent electrical circuit, and (**B**) variation of the R_CT_ with the logarithmic concentration of *S. aureus*.

In order to express the characterization of modified electrode/electrolyte interface after the immobilization of biomolecules and bacteria binding, EIS is often analyzed using an equivalent circuit. The equivalent circuit is applied to fit the experimental data and extract the necessary information about the electrical parameters responsible for the impedimetric change. The impedance spectra obtained in the presence of different concentrations of bacteria ranging from 10 to 10^7^ CFU/mL were modeled by the equivalent electrical circuit shown in Figure 6(A). The circuit is a combination of:

Series resistance of solution and of ohmic contacts (Rs).

- A resistance related to the charge transfer rate of the redox reaction at the biofunctionalized electrode; R_CT_.
- A constant phase element (CPE) that is related to the capacitance of the biofunctionalized Au electrode/electrolyte interface. CPE reflects the non-ideality of the double-layer at the biofunctionalized gold electrode/electrolyte interface due to the roughness and porosity of the biofilm.
- A specific electrochemical element of diffusion, the Warburg element (Zw).

The impedance immunosensor shows a linear relationship between the R_CT _variation and the logarithmic value of *S. aureus* concentrations. These were found within the range of 10 to 10^6 ^CFU/mL with a correlation coefficient of 0.994. The variation of R_CT_ was calculated against the value of R_CT_ in the absence of bacteria (Figure 6(B)). The reproducibility of the immunosensor was validated by repeating the experiment with four different immunosensors prepared in the same conditions. The results showed that the response of the immunosensor has an average standard deviation of 8%. This verifies that a good reproducibility of the system was achievable for the detection of S. aureus.

**Figure 7:**
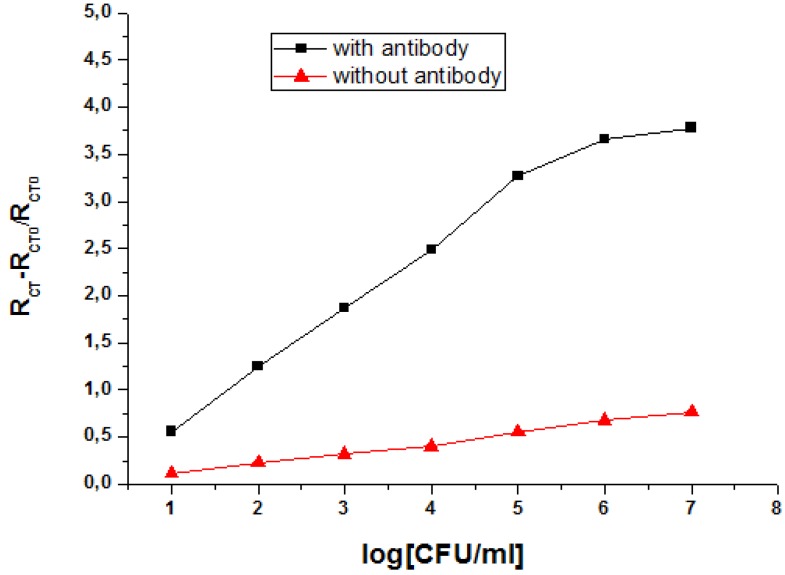
A comparative study of the response of the modified electrode (relative variation of R_CT_) in the presence and absence of antibodies.

A comparative study was made to ensure that the response of the immunosensor was a direct cause of the immobilized antibody and not due to adsorption of bacteria on the surface modified SAMs. Here, different electrodes were functionalized in the same conditions as our immunosensor, but without the antibody incubation step. The results show that there is not a significant variation of the response for the modified electrode (relative variation of R_CT_) after incubation of the electrode in varying *staphylococcus* concentrations (Figure 7). The fixation of the antibody is a logical approach for the high detection of these pathogenic bacteria.

The characteristics of relevant and previous immunosensors for *S. aureus* detection in terms of the limit of detection (LOD) and of the dynamic range are given in Table 1. In this work, the LOD obtained for the developed immunosensor is of the lower value and the dynamic range is of the larger value.

**Table 1:** A comparison of the analytical characteristics of the immunosensor developed in this work with relevant immunosensors for *S. aureus* bacteria detection based in the literature.

Type of transducer	Detection Limit	Dynamic range	Reference
Impedance	10 CFU/mL	10–10^6^ CFU/mL	This work
Impedance	10^2^ CFU/mL	10^2^–10^7^ CFU/mL	[11]
Amperometry	3.7 × 10^2^ CFU/mL	1.3 × 10^3^–7.6 × 10^4^ CFU/mL	[10]
SPR	10^5^ CFU/mL	-	[9]

**Figure 8:**
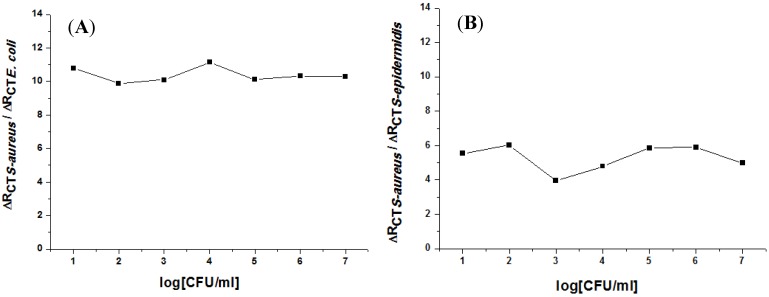
Positive-to-negative response ratio of the developed immunosensor for (**A**) *E. coli*, and (**B**) *S. epidermidis* bacteria.

*E. coli *and *Staphylococcus epidermidis (S. epidermidis)* were detected in order to determine the specificity of our impedimetric immunosensor. These two bacteria are the most abundant strains found in real samples. The same protocol was followed for the surface functionalization using anti-*S. aureus*. Here, the antibody was immobilized and the electrodes were exposed to the suspensions of *E. coli *and *S. epidermidis*. In a similar manner to the detection of *S. aureus*, the concentrations of the two bacteria were increased in order to observe if there was a significant effect on the immunosensor by EIS. In Figure 8, the positive-to-negative response ratios of the immunosensor for non-specific targets (*E. coli* and *S. epidermidis*) are given. The results show a higher selectivity for *E. coli* compared to *S. epidermidis*.

## 4. Conclusion

In this work, our immunosensor was monitored step by step; the surface functionalization by SAMs, the antibody binding, and pathogenic bacteria detection. The performance of this sensor was evaluated in terms of its sensitivity, limit of detection, dynamic range, reproducibility, and specificity for *S. aureus *bacteria. The sensitivity of 127 Ω/decade, the detection limit of 10 CFU/mL, and a correlation coefficient of 0.994 was obtained with a good reproducibility (average standard deviation of 8%). A negative test was performed by injecting several concentrations of non-specific targets; *E. coli *and *S. epidermidis *bacteria. The absence of any significant change confirms the specificity of the developed immunosensor as only *S. aureus* was detected. The only drawback is that our immunosensor is not reusable, since we were not able to regenerate our antibodies for subsequent detections.
